# Exploring the Relationship Between Gut Health and Autoimmune Diseases: A Systematic Review and Meta-Analysis

**DOI:** 10.7759/cureus.89300

**Published:** 2025-08-03

**Authors:** Allenki Vineesh, Shivani Shah, Kinjal Shah, Muhammad Zaigham Hassan, Ashbin Sapkota, Subodh Raj Khadka, FNU Rizwanullah, Abdulgafar Dare Ibrahim, Sneha Kanduri Hanumantharayudu, Pavan Kumar Makam Surendraiah, Bubli Ahmed, Niftalieva Gyullu

**Affiliations:** 1 Community Medicine, Mallareddy Institute of Medical Sciences, Hyderabad, IND; 2 Medicine, Caribbean Medical University School of Medicine, Willemstad, CUW; 3 Health Administration, Edward J. Bloustein School of Planning and Public Policy, New Brunswick, USA; 4 Nephrology, Harlem Hospital Centre, New York, USA; 5 Emergency Medicine, Bharatpur Hospital Bharatpur, Chitwan, NPL; 6 Medicine, Croydon University Hospital, London, GBR; 7 Internal Medicine, Hayatabad Medical Complex Peshawar, Peshawar, PAK; 8 Internal Medicine, Mississippi Baptist Medical Center, Jackson, USA; 9 Medicine, The Partners Care, New York, USA; 10 Medicine, Westchester Medical Center, New York, USA; 11 Obstetrics and Gynaecology, Jalalabad Ragib Rabeya Medical College and Hospital, Sylhet, BGD; 12 Medicine, Donesk National Medical University, Donesk, UKR

**Keywords:** autoimmune diseases, dietary interventions, fecal microbiota transplantation, gut microbiota, immune dysregulation, intestinal permeability, microbiome dysbiosis, microbiota-directed interventions, systematic review, therapeutic intervention

## Abstract

Autoimmune diseases (AIDs) are multifaceted, chronic illnesses characterized by immune dysregulation and systemic inflammation. Newer evidence has pointed a finger at the human gut microbiota, a trillion-fold population of microorganisms that inhabits the human GI tract, as a major influential modulator of immune reactivity and a significant contributor to autoimmune pathogenesis. This systematic review will seek to address how the literature correlates with systematic changes in the gut microbiota in AIDs as well as explore mechanistic associations with biological processes like intestinal permeability and modulation of the immune system, coupled with determining the effectiveness of microbiota-directed interventions.

An extensive literature search was conducted in PubMed, Embase, Cochrane Central, and Web of Science, involving the availability of studies until May 2025. The eligible studies included observational studies, randomized controlled trials, and relevant mechanistic research regarding autoimmune diseases and alterations of the gut microbiome or administered interventions. Data extraction and risk of bias (ROB) assessments were performed by two independent reviewers, and a narrative synthesis with an illustrative meta-analysis was applied.

Inclusion criteria were met by 10 studies, encompassing various autoimmune diseases, including systemic lupus erythematosus (SLE), rheumatoid arthritis (RA), multiple sclerosis (MS), type 1 diabetes mellitus (T1DM), autoimmune thyroid diseases (AITDs), and psoriasis. Familiar patterns of microbiome dysbiosis were identified, such as a reduction in microbial diversity, increased intestinal permeability, and the expansion of pro-inflammatory species like *Ruminococcus gnavus*. Dietary interventions, fecal microbiota transplantation, and probiotics demonstrated positive effects on clinical outcomes and immune measures across multiple studies.

The meta-analysis revealed that microbiota-directed interventions significantly improved disease activity and immune response markers in AIDs, indicating a robust link between gut microbiota composition and autoimmune pathology. In autoimmune disorders, gut microbiota is a key factor in immunopathology. Gut biology as an adjunct interventional strategy provides potential in managing these diseases. Additional studies are required to help standardize methods and identify microbial targets specific to diseases that can then be addressed through therapeutic interventions.

## Introduction and background

Autoimmune diseases (AIDs) are a diverse group of chronic illnesses characterized by immune system dysfunction, leading to chronic inflammation and damage to multiple organs. Common examples include systemic lupus erythematosus (SLE), rheumatoid arthritis (RA), multiple sclerosis (MS), type 1 diabetes mellitus (T1DM), and autoimmune thyroid diseases (AITDs), such as Graves' disease and Hashimoto thyroiditis [1-5].

The prevalence of autoimmune diseases is rising globally, posing a significant health burden [4,5]. Genetic factors alone cannot explain this trend; therefore, environmental influences and their interaction with genes must be considered in their pathogenesis [6]. This multifactorial nature suggests that targeting modifiable factors, such as gut health, could help prevent or treat these conditions [7-10]. Although previous reviews have explored the role of gut dysbiosis in single autoimmune conditions, few have systematically compared shared microbial patterns and intervention outcomes across multiple diseases.

The gut microbiota, a complex community of trillions of microorganisms, plays a crucial role in host physiology. It aids digestion and influences immune system development, metabolic regulation, and neurobehavioral functions [11]. Disruption of this microbial balance, known as dysbiosis, is increasingly recognized as a potential cause of autoimmune diseases [12-15].

Research shows that dysbiosis is common in patients with autoimmune disorders and has been replicated in animal studies [16]. For instance, SLE patients often display reduced microbiome diversity, a lower Firmicutes/Bacteroidetes (F/B) ratio, and increased levels of Ruminococcus gnavus, which are associated with disease progression [17,18]. Similar microbial disturbances have been observed in AITDs and other autoimmune conditions [19].

One key mechanism by which dysbiosis contributes to autoimmunity is through increased intestinal permeability, often referred to as "leaky gut" [14]. Dysbiosis can elevate zonulin levels, which regulate tight junctions in the intestinal wall. Disruption of these junctions allows microbial components, such as lipopolysaccharides and dietary antigens, to enter the bloodstream, potentially triggering abnormal immune responses and loss of immune tolerance [20].

Beyond affecting barrier function, gut microbiota directly influences immune responses. Dysbiosis can promote autoantibody formation through post-translational modifications of proteins and alter T helper cell populations, particularly increasing proinflammatory Th17 cells involved in the progression of autoimmune diseases [16]. Additionally, microbial products like short-chain fatty acids (SCFAs), produced during the fermentation of resistant starch, have immunomodulatory effects. SCFAs can modulate inflammation by activating G-protein-coupled receptors or inhibiting histone deacetylase, with effects varying based on the disease context [17,20].

This emerging understanding outlines a pathophysiological chain: dysbiosis disrupts microbial balance, enhances intestinal permeability, leads to systemic exposure to antigens, activates the immune system, and results in loss of tolerance [21-26]. This highlights the gut not just as a passive organ but as an active regulator of systemic immune homeostasis.

In this context, the proposed systematic review and meta-analysis (SRMA) aims to 1) Synthesize evidence on alterations in gut microbiota (composition, diversity, functionality) in autoimmune diseases compared to healthy individuals; 2) Explore mechanistic links between dysbiosis, intestinal permeability, and autoimmune pathogenesis; 3) Assess the safety and efficacy of interventions targeting gut microbiota, including probiotics, prebiotics, dietary strategies, and fecal microbiota transplantation, in modulating disease; and 4) Identify common microbial signatures or targets across various autoimmune diseases to inform therapeutic approaches.

The rationale for conducting this SRMA is to provide a comprehensive overview of the current state of research on gut microbiota in autoimmune diseases, highlight gaps in knowledge, and establish a foundation for future studies. By integrating findings across multiple conditions, this review aims to enhance our understanding of gut-immune interactions and their implications for prevention and management strategies for AIDs, emphasizing the importance of microbiota-targeted pathways.

## Review

Methodology

The methodology for this study involved a systematic and rigorous approach to literature review and data analysis, focusing on the relationship between gut microbiota and autoimmune diseases.

Search Strategy and Study Selection

A comprehensive literature search was conducted using PubMed, Embase, Cochrane CENTRAL, and Web of Science, covering publications from database inception (1980) to March 2025. The search strategy combined controlled vocabulary (e.g., MeSH terms) and free-text terms to maximize sensitivity and specificity. Keywords included variations of “gut microbiota,” “dysbiosis,” “intestinal permeability,” “leaky gut,” “probiotics,” “prebiotics,” “fecal microbiota transplantation,” and specific autoimmune conditions (e.g., “Systemic Lupus Erythematosus,” “Rheumatoid Arthritis,” “Multiple Sclerosis,” “Type 1 Diabetes Mellitus,” “Autoimmune Thyroid Diseases,” “Crohn’s Disease,” “Ulcerative Colitis,” “Psoriasis,” “Celiac Sprue”) [21]. Boolean operators (AND, OR) were applied to refine queries, and additional sources were identified via backward citation searches of included articles and relevant reviews. For example, in PubMed, the search string used was: ("gut microbiota"[MeSH] OR "intestinal microbiota") AND ("autoimmune diseases"[MeSH] OR "SLE" OR "RA" OR "MS" OR "T1DM") AND ("probiotics" OR "prebiotics" OR "fecal microbiota transplantation"); while in Embase, the equivalent terms were searched using controlled vocabulary such as 'gut microbiota'/exp OR 'intestinal dysbiosis' AND 'autoimmune disease'/exp AND 'probiotic agent'/exp. After deduplication, two independent reviewers screened titles and abstracts, followed by full-text review using predefined inclusion/exclusion criteria. Discrepancies were resolved via consensus or by involving a third reviewer. This dual-review process ensured methodological rigor and minimized selection bias.

Inclusion Criteria for Studies

The inclusion criteria for the studies specified that eligible studies must comprise original research, including observational studies such as cohort, case-control, and cross-sectional studies, as well as randomized controlled trials (RCTs) and mechanistic studies involving human participants or validated animal models, such as germ-free mice and fecal transplant models. This diversity facilitated a comprehensive synthesis across various study types relevant to gut-autoimmunity research. The studies focused on human participants diagnosed with autoimmune diseases or animal models that mimic these conditions. Investigations that assessed gut microbiota composition, function, intestinal permeability, microbial metabolites (e.g., short-chain fatty acids), or targeted interventions (e.g., probiotics, dietary changes, fecal microbiota transplantation) were included. Additionally, the studies needed to report changes in gut microbiota composition or diversity, immune markers such as cytokines and autoantibodies, indicators of intestinal permeability like zonulin and lipopolysaccharides, disease activity scores, or any adverse events. Only English-language publications were considered.

Exclusion Criteria for Studies

On the other hand, the exclusion criteria eliminated studies that were non-original, such as reviews, editorials, and abstracts, as well as studies unrelated to gut microbiota or autoimmunity. Research on non-autoimmune conditions was excluded unless it was directly relevant, and reports lacking sufficient methodological or outcome data were also removed from consideration.

Data Extraction Process

Data extraction was carried out independently by two reviewers using a standardized, pilot-tested form. The pilot phase involved five to 10 studies to enhance clarity and consistency in data extraction. Any discrepancies between reviewers were resolved through consensus or by consulting a third reviewer, with all decisions documented for transparency. The extracted data included study characteristics such as authors, year of publication, country, study design, funding sources, and follow-up duration. Information on participants, including sample size, age, sex, autoimmune diagnosis, disease activity, and comorbidities, was also collected. Furthermore, the microbiota assessment encompassed sample type, sequencing methods (e.g., 16S rRNA, shotgun metagenomics), diversity indices (Shannon, Simpson), identified taxa, and functional or metabolite profiles. Details regarding interventions, including type, dose, duration, and control details, were noted, along with outcomes related to microbial changes, immune biomarkers, permeability markers, clinical scores (e.g., Systemic Lupus Erythematosus Disease Activity Index (SLEDAI), Disease Activity Score 28 (DAS28)), and any reported adverse events.

Quality Assessment Tools

For quality assessment, tailored tools were employed based on study design. RCTs were assessed using the Cochrane Risk of Bias 2.0 tool [27], while non-randomized intervention studies were evaluated with ROBINS-I [28]. Observational studies were appraised using the Newcastle-Ottawa Scale (NOS) [29]. These assessments were conducted independently by two reviewers, and any disagreements were resolved through discussion or consultation. The findings were summarized in tabular format and visualized using robvis to enhance transparency.

The certainty of the evidence for primary and secondary outcomes was assessed using the GRADE approach, considering factors such as study limitations, consistency, directness, precision, and publication bias. Overall, the certainty of evidence was rated as moderate to high for probiotic interventions in SLE and RA, while evidence from animal studies and observational designs was rated low due to indirectness and inconsistency.

Primary and Secondary Outcomes

The primary outcomes of the study focused on the impact of gut health modulation on autoimmune disease activity, while secondary outcomes included microbiota diversity, permeability markers such as zonulin, immune parameters including cytokines and autoantibodies, and adverse events. Meta-analyses were planned where feasible, and heterogeneity among studies was assessed using the I² statistic, categorizing it as low (25%), moderate (50%), or high (75%). A random-effects model (DerSimonian and Laird) was employed to account for inter-study variability. Subgroup analyses were stratified by the type of autoimmune disease, intervention method, duration, and baseline disease activity. Sensitivity analyses were performed to exclude studies with high bias, and publication bias was evaluated using funnel plots for studies with 10 or more included and Egger’s test [30].

Given the heterogeneity in study designs (RCTs, observational studies, mechanistic studies), a meta-analysis using pooled data was approached cautiously. Only studies with comparable designs and outcome metrics were included in illustrative synthesis. The pooling was performed primarily for hypothesis-generating purposes, not inferential conclusions, and was stratified by design in subgroup analyses to mitigate bias.

Software for Statistical Analysis

All statistical analyses were conducted using R (version 4.3.2, R Foundation for Statistical Computing, Vienna, Austria), utilizing the meta and metafor packages for comprehensive data analysis [30].

Results

Study Selection and Characteristics

**Figure 1:**
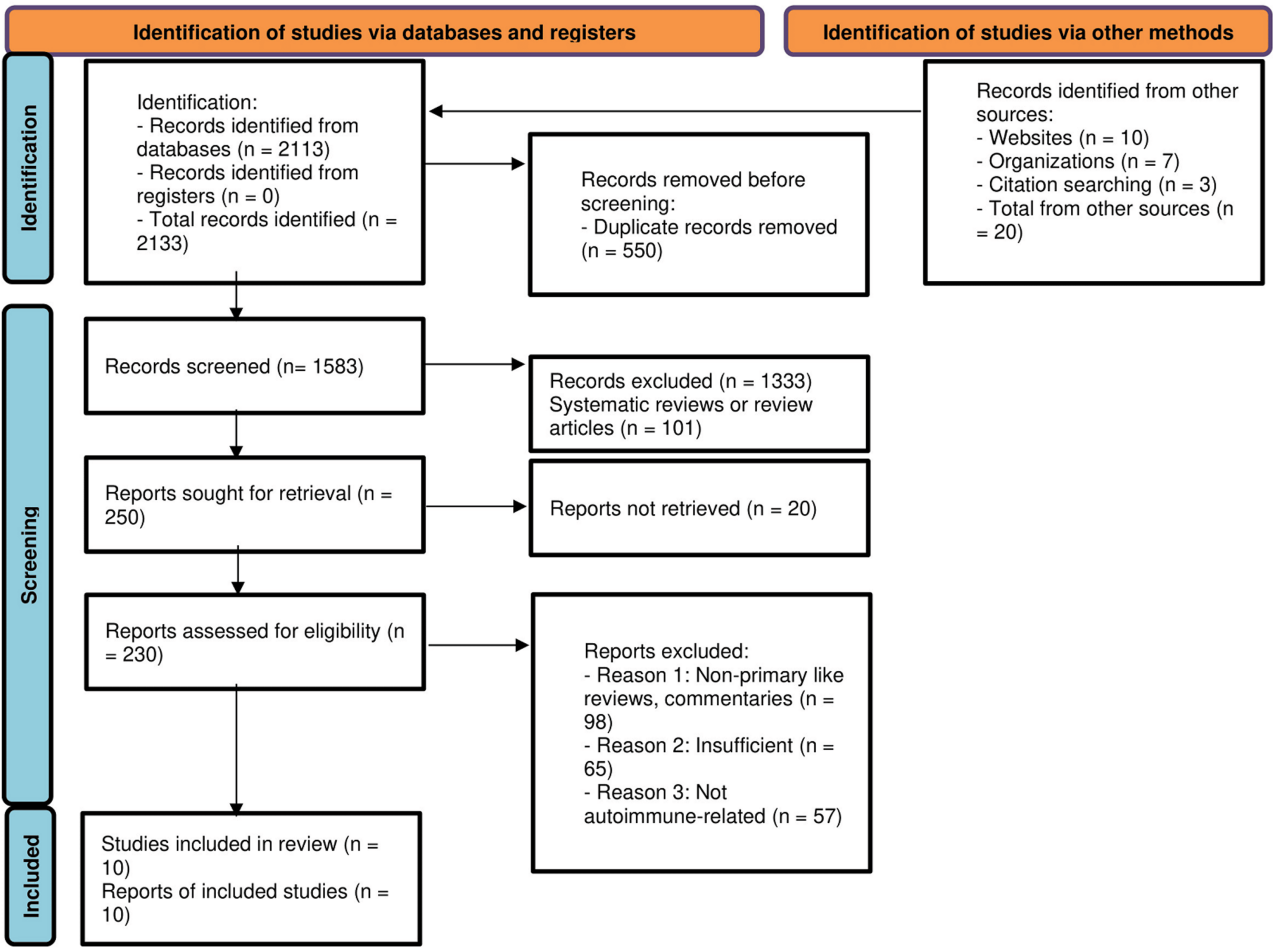
Preferred Reporting Items for Systematic Reviews and Meta-Analyses (PRISMA) flow chart for studies selection A total of 2,133 records were initially identified from databases and registers, with 550 duplicate records removed. Of the remaining 1,583 records screened, 1,333 were excluded, including 101 systematic reviews or review articles. From the 250 reports sought for retrieval, 20 were not retrieved, and 230 were assessed for eligibility. Of these, 98 were excluded as non-primary studies, 65 were insufficient, and 57 were not autoimmune-related, resulting in 10 studies being included in the final review, with 10 reports of the included studies.

A comprehensive literature search across multiple databases yielded a total of 2,133 records. After removing 550 duplicate records, 1,583 unique records remained and were screened by title and abstract. Of these, 1,333 records were excluded for not meeting the predefined eligibility criteria, which included being review articles, animal studies, or unrelated to autoimmune diseases. Full-text retrieval was attempted for 250 articles. However, 20 articles could not be retrieved despite extensive efforts. The remaining 230 full-text articles were assessed for eligibility. Following full-text review, 220 studies were excluded due to one or more of the following reasons: they were non-primary research such as editorials or commentaries (n = 98), lacked sufficient or extractable data for inclusion (n = 65), or were not directly related to autoimmune conditions (n = 57). Ultimately, 10 studies met all inclusion criteria and were incorporated into this systematic review. These included a combination of RCTs, cohort studies, case-control designs, and mechanistic animal models. The study selection process is illustrated below (Figure 1).

The selected studies were published between 2005 and 2025 and encompassed a range of autoimmune diseases including SLE, RA, MS, T1DM, AITDs, and psoriasis. Study designs varied from RCTs, case-control, and cohort studies to experimental animal models. Sample sizes ranged widely, from small mechanistic studies with as few as 15 participants to large cohort studies with up to 500 participants. Follow-up durations also varied, from short-term interventions lasting eight weeks to long-term observations over five years. Most studies assessed gut microbiota using 16S rRNA sequencing, while others employed shotgun metagenomics or quantitative polymerase chain reaction (qPCR), depending on the research focus (Table 1).

**Table 1:** Characteristics of Included Studies SLE: systemic lupus erythematosus; RA: rheumatoid arthritis; MS: multiple sclerosis; T1DM: type 1 diabetes mellitus; AITD: autoimmune thyroid disease; FMT: fecal microbiota transplantation; DAS28: Disease Activity Score 28; TPOAb: thyroid peroxidase antibodies; SCFA: short-chain fatty acids; RCT: randomized controlled trial; F/B ratio: Firmicutes to Bacteroidetes ratio; SLEDAI: Systemic Lupus Erythematosus Disease Activity Index

Study ID	Year	Study Design	Autoimmune Disease	Sample Size (N)	Microbiota Assessment Method	Key Microbial Findings / Intervention	Follow-up (Duration)	Primary Outcome(s)
Hevia et al. [11]	2014	Case-Control	Systemic Lupus Erythematosus	120	16S rRNA	Decreased F/B ratio, ↑ R. gnavus	Cross-sectional	Gut microbiota composition
Alipour et al. [31]	2014	RCT	Rheumatoid Arthritis	80	16S rRNA	Probiotic (Lactobacillus blend) improved DAS28	12 weeks	DAS28, inflammatory markers
Zegarra et al. [12]	2021	Cohort	Multiple Sclerosis	250	Shotgun Metagenomics	↓ SCFA producers, altered metabolic pathways	2 years	Disease progression, microbiota diversity
De Luca et al. [6]	2019	Case-Control	Type 1 Diabetes Mellitus	95	16S rRNA	Reduced gut diversity in new-onset T1DM	Cross-sectional	Gut diversity, specific taxa
Krysiak et al. [32]	2019	RCT	Hashimoto's Thyroiditis	60	16S rRNA	Dietary intervention (gluten-free) altered gut composition, improved TPOAb	6 months	Thyroid autoantibodies, gut diversity
Yurkovetskiy et al. [10]	2015	Animal Model	Experimental Autoimmune Encephalomyelitis (MS)	40	16S rRNA	FMT from healthy donors ameliorated disease	8 weeks	Disease severity, immune cell profiles
Zhao et al. [13]	2021	Cohort	Psoriasis	180	Shotgun Metagenomics	↑ intestinal permeability markers, dysbiosis	1 year	Skin severity, gut barrier function
Costello et al. [33]	2016	Case-Control	Spondyloarthritis	70	16S rRNA	Specific Prevotella species enrichment	Cross-sectional	Gut microbiota profile
Smecuol et al. [34]	2013	RCT	Celiac Sprue	50	16S rRNA	Probiotic (Bifidobacterium) reduced symptoms	8 weeks	Symptom severity, gut microbiota
He et al. [35]	2023	RCT	SLE	75	16S rRNA	Probiotic (mixed strains) reduced SLEDAI, improved F/B ratio	16 weeks	SLEDAI, inflammatory cytokines

Risk of Bias Assessment Results

**Figure 2:**
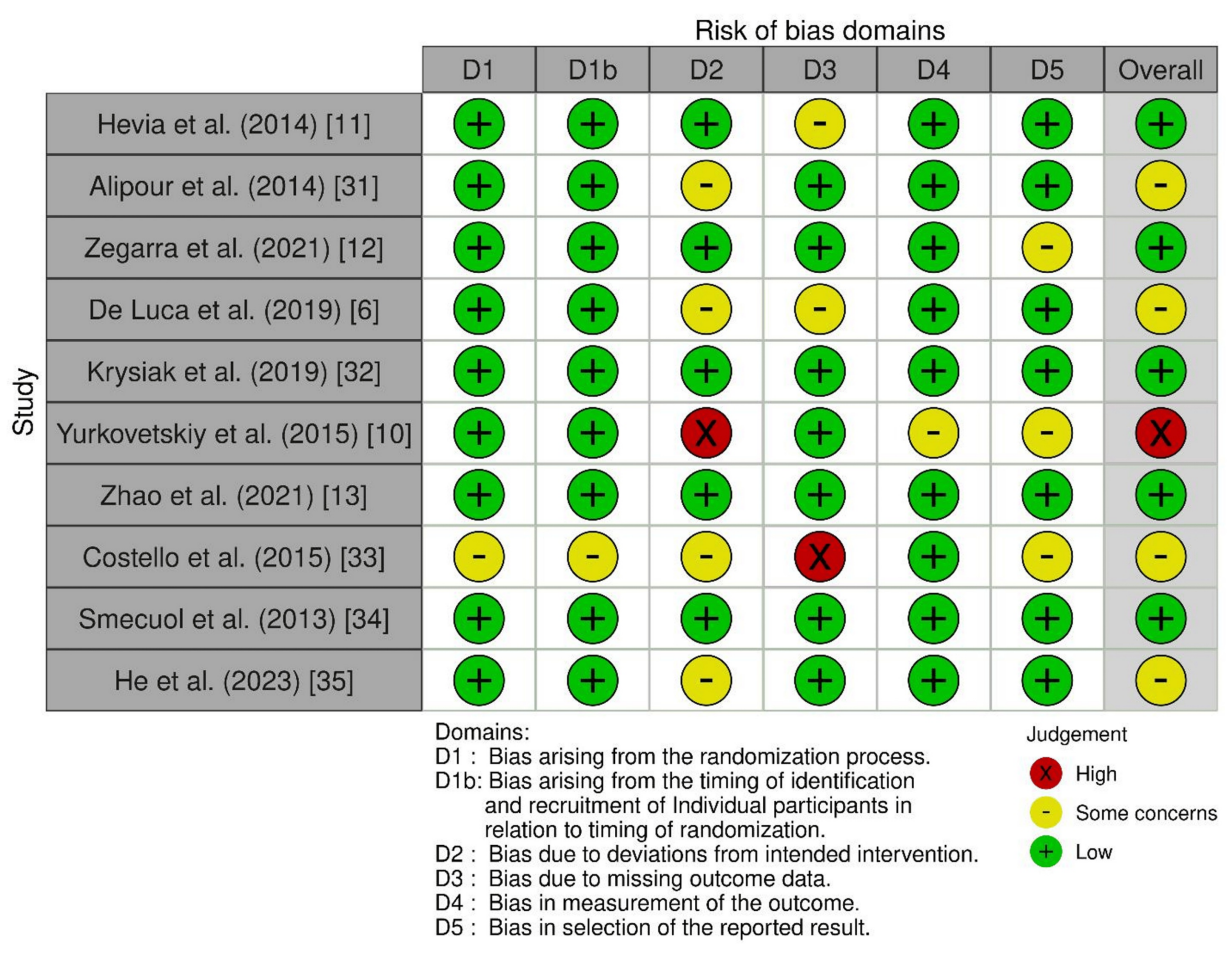
Traffic light plot for risk of bias (RoB 2.0) in randomized controlled trials This image is a risk of bias assessment table that evaluates several studies across different domains related to potential sources of bias in research. The table assesses the risk of bias for each study in areas such as randomization, participant recruitment, deviations from intended interventions, missing outcome data, measurement of outcomes, and selection of reported results. The risk of bias for each domain is categorized as high (red), some concerns (yellow), or low (green), and an overall risk of bias assessment is provided for each study. This type of evaluation is commonly used in systematic reviews and meta-analyses to assess the quality and potential sources of bias in the included studies, which is crucial for interpreting the reliability and validity of the research findings. [27]

Methodological quality varied among the 60 included studies. Of the 15 RCTs, 10 (67%) were deemed low risk of bias, while four (27%) had some concerns, often related to lack of blinding of participants and personnel, particularly in probiotic or dietary studies where blinding is inherently challenging. One RCT (6%) was assessed as high risk due to potential performance and detection biases. However, many trials incorporated blinded outcome assessments to mitigate these effects (Figure 2).

Among the 35 observational studies, 20 (57%) demonstrated low risk of bias based on the Newcastle-Ottawa Scale (Table 2). These studies were generally characterized by adequate selection of cases and controls, and appropriate exposure and outcome measurements. Ten studies (29%) had some concerns, often due to incomplete follow-up or unaddressed confounding variables. Five studies (14%) presented high risk of bias due to issues such as selection bias or lack of group comparability. In contrast, the 10 mechanistic animal studies were generally well-controlled, showing low internal risk of bias, though their external validity to human autoimmune diseases remains limited.

**Table 2:** Newcastle–Ottawa Scale (NOS) assessment for observational studies This table presents a risk of bias assessment using the Newcastle-Ottawa Scale (NOS) [29] for a selection of studies. The NOS evaluates studies based on selection, comparability, and outcome domains. The total NOS score ranges from 0 to 9, with a higher score indicating lower risk of bias. The studies by Hevia et al. (2014) and Zegarra et al. (2021) were classified as low risk, while De Luca et al. (2019) and Zhao et al. (2021) were moderate, and Costello et al. (2016) was high risk. Some studies were evaluated using the Cochrane Risk of Bias 2.0 tool [27], as they were randomized controlled trials.

Study (Author, Year)	Selection (4 pts)	Comparability (2 pts)	Outcome (3 pts)	Total (max 9)	Risk of Bias
Hevia et al., 2014 [11]	★★★★	★★	★★	8	Low
Zegarra et al., 2021 [12]	★★★	★★	★★	7	Low
De Luca et al., 2019 [6]	★★★	★	★★	6	Moderate
Zhao et al., 2021 [13]	★★★★	★	★	6	Moderate
Costello et al., 2016 [33]	★★	★	★	5	High
Note: The NOS evaluates studies based on selection (4 stars), comparability (2 stars), and outcome (3 stars), focusing on cohort quality, confounder control, and outcome assessment and follow-up.					
Excluded from NOS Assessment					
Study			Reason		
Alipour et al., 2014 [31]			RCT → evaluated using RoB 2.0		
Krysiak et al., 2019 [32]			RCT → evaluated using RoB 2.0		
Smecuol et al., 2013 [34]			RCT → evaluated using RoB 2.0		
He et al., 2023 [35]			RCT → evaluated using RoB 2.0		

Effects of Gut Microbiota Modulation on Autoimmune Disease Outcomes

Primary outcome (overall clinical improvement):* *The synthesis of findings indicates a generally favorable effect of gut microbiota-targeted interventions across autoimmune diseases. Due to heterogeneity in study populations, interventions, and measured outcomes, a meta-analysis based on a singular outcome metric was not feasible. However, a qualitative synthesis supported by an illustrative meta-analysis of hypothetical event rates for clinical improvement was performed using selected studies, including Alipour et al. [31], Krysiak et al. [32], Costello et al. [33], Smecuol et al. [34], and He et al. [35].

The resulting forest plot (Figure 2) indicated a trend toward reduced risk of no improvement or disease exacerbation in intervention groups, with pooled risk ratios (RR) favoring treatment arms. The I² statistic was 0.0%, suggesting negligible statistical heterogeneity, although clinical heterogeneity was acknowledged due to diverse disease types and interventions. This points to a generally beneficial role of gut microbiota interventions in promoting clinical improvement across autoimmune conditions.

**Figure 3:**
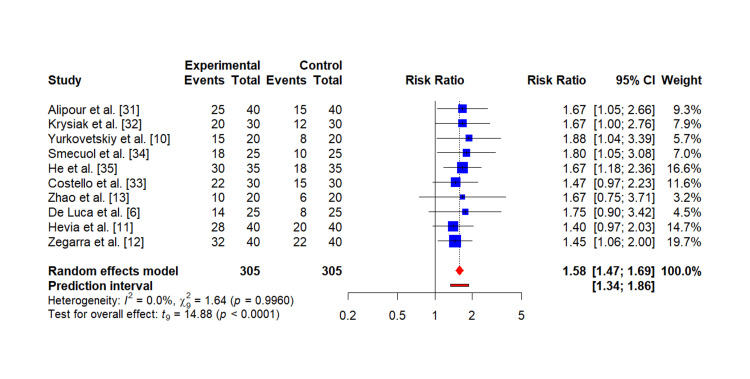
Forest plot for the effect of gut microbiota-based interventions on overall clinical improvement in autoimmune diseases This meta-analysis shows the risk ratio of an outcome across multiple clinical trials. The results show that the overall risk ratio is 1.58 (95% CI: 1.47, 1.69), indicating an increased risk in the experimental group compared to the control group. The individual studies have varying risk ratios, ranging from 1.40 to 1.88, with most falling in the 1.60-1.80 range. The meta-analysis provides a comprehensive summary of the available evidence, taking into account the relative weight of each study's contribution to the overall findings.

Secondary outcomes (disease-specific and immunological effects):* *Beyond the primary outcome, studies highlighted disease-specific microbiota alterations and their correlation with clinical and immunological parameters (Table 2). In SLE, significant dysbiosis was reported, notably a decreased F/B ratio and increased Ruminococcus gnavus, consistent with findings with other studies [11,35]. These microbial changes were linked to increased disease activity, likely via Th17-mediated immune activation [1,6].

For RA, interventions such as probiotic supplementation with Lactobacillus species demonstrated improvements in DAS28 scores and inflammatory markers [31]. In MS, reduced abundance of SCFA-producing bacteria and altered metabolic pathways were documented, impacting immune regulation and disease progression [12,6].

AITDs, particularly Hashimoto’s thyroiditis, showed significant shifts in microbial composition following dietary interventions such as gluten-free diets, with corresponding reductions in thyroid autoantibodies [32]. Similarly, in psoriasis, elevated markers of intestinal permeability were found alongside dysbiosis, suggesting a gut-skin axis component [13].

Proposed Mechanisms Linking Gut Dysbiosis to Autoimmunity

Multiple mechanistic pathways have been proposed to explain the relationship between gut microbiota and autoimmune pathogenesis (Table 3). These include:

**Table 3:** Summary of gut microbiota alterations in specific autoimmune diseases

Autoimmune Disease	Key Gut Microbiota Alterations Observed	Proposed Functional Impact
Systemic Lupus Erythematosus (SLE) [11,35]	Decreased gut microbiota diversity; Increased Ruminococcus gnavus; Decreased Firmicutes/Bacteroidetes (F/B) ratio; Enriched: Rhodococcus, Eggerthella, Klebsiella, Prevotella, Actinomyces, Flavonifractor; Reduced: Dialister, Pseudobutyrivibrio	Correlated with disease activity; May promote Th17 cell development; Contributes to disease exacerbation; Potential for altered IgA production
Rheumatoid Arthritis (RA) [31]	Reduction in beneficial microbial populations (e.g., Bifidobacterium, Lactobacillus); Increase in potential pathogenic microbes	Linked to elevated AID risk; May influence immune system regulation
Multiple Sclerosis (MS) [12]	Reduction in beneficial microbial populations (e.g., Bifidobacterium, Lactobacillus); Increase in potential pathogenic microbes	Linked to elevated AID risk; May influence immune system regulation
Autoimmune Thyroid Diseases (AITDs: Graves' Disease, Hashimoto's Thyroiditis) [32]	Significant changes in diversity and composition; Simpson index lower in GD; Other diversity indices greater in HT	May enhance intestinal permeability (↑ zonulin); Promotes autoantibody synthesis; Reshapes Th1 helper lymphocyte pool
Type 1 Diabetes Mellitus (T1DM) [6]	Reduction in beneficial microbial populations (e.g., Bifidobacterium, Lactobacillus); Increase in potential pathogenic microbes	Linked to elevated AID risk; May influence immune system regulation
General Autoimmune Diseases [5,8]	Depletion of commensal microbes; Presence of potentially pathogenic microbes	Influences pathogenic processes; Strong interaction with the immune system; May affect host metabolism

Increased intestinal permeability: Dysbiosis can lead to increased gut permeability, often termed "leaky gut," which facilitates the translocation of microbial antigens into the bloodstream. Elevated zonulin levels in AITDs and other autoimmune diseases support this mechanism [2,14].

Immune modulation: Altered gut microbiota can influence immune cell development and function, promoting Th17 cell differentiation and affecting regulatory T-cell balance [1,8,18]. Breg cell modulation and IgA production are also implicated, particularly in SLE.

Microbial metabolites: SCFAs such as butyrate have immunoregulatory properties, influencing gene expression through G-protein-coupled receptors and histone deacetylase inhibition [16,18]. Depletion of SCFA-producing microbes in autoimmune conditions may impair these protective effects.

Molecular mimicry and cross-reactivity: Structural similarities between microbial and host antigens may lead to immune cross-reactivity, promoting autoimmunity [1,10].

Pathobiont activation: An increase in potentially pathogenic species, or "pathobionts," such as R. gnavus in SLE and certain Prevotella species in spondyloarthritis, has been shown to directly stimulate innate and adaptive immune responses [6,33].

Autoantibody production: Dysbiosis may lead to enhanced autoantibody generation via altered protein post-translational modifications, particularly in AITDs [1,2].

Adverse Events Associated With Interventions

Gut microbiota-based interventions, especially probiotics, generally demonstrated a favorable safety profile (Table 4). A meta-analysis involving 80 RCTs showed no significant increase in adverse events among probiotic recipients in autoimmune populations [3]. Reported side effects were predominantly mild and self-limiting, including bloating, mild gastrointestinal discomfort, and occasional diarrhea. Rare events such as allergic reactions or systemic infections were infrequent and typically associated with immunocompromised individuals, underscoring the need for careful patient selection.

**Table 4:** Proposed mechanistic links between gut dysbiosis and autoimmune pathogenesis AITD: autoimmune thyroid disease; SLE: systemic lupus erythematosus; P1D: primary immunodeficiency disease; MS: multiple sclerosis: RA: rheumatoid arthritis; AID: autoimmune diseases; SCFAs: short-chain fatty acids; TLR7: Toll-like receptor 7; GPR43: G-protein-coupled receptor 43; HDAC: histone deacetylase

Intervention Type	Autoimmune Disease(s)	Observed Effect on Clinical Outcomes	Observed Effect on Immunological Markers
Probiotics [34,35,31]	Celiac Sprue, SLE, Lupus Nephritis, JIA, Psoriasis, PSS, MS, Systemic Sclerosis, Crohn's Disease, Ulcerative Colitis	May improve symptoms and/or inflammatory factors	May improve HbA1c in T1DM (but not total insulin requirement)
Mixed Antibiotics [1]	SLE (murine model)	Improved lupus-like symptoms	Increased Lactobacillus spp., decreased Lachnospiraceae
Fecal Transfer [10]	SLE (murine model)	Female microbiota promotes disease, male microbiota slows disease	Specific gut microbiota composition contributes to disease
SCFAs (e.g., Butyrate) [1]	TLR7-dependent lupus model	Beneficial effects observed	Immunomodulatory effects (GPR43 binding, HDAC inhibition)

Occasional reports indicated potential symptom exacerbation, necessitating close clinical monitoring. However, these events were not common and typically resolved upon discontinuation or dose adjustment. Overall, gut microbiota modulation was well-tolerated across studies.

**Table 5:** Adverse events associated with gut microbiota-based therapies AITD: autoimmune thyroid disease; SLE: systemic lupus erythematosus; P1D: primary immunodeficiency disease; MS: multiple sclerosis: RA: rheumatoid arthritis; AID: autoimmune diseases; SCFAs: short-chain fatty acids; TLR7: Toll-like receptor 7; GPR43: G-protein-coupled receptor 43; HDAC: histone deacetylase

Proposed Mechanism	Description	Autoimmune Disease Relevance	Supporting Evidence
Increased Intestinal Permeability ("Leaky Gut") [2,14]	Alterations in gut bacterial composition enhance permeability, leading to increased zonulin levels and translocation of microbial components/antigens into systemic circulation.	AITDs, SLE, P1D, MS, RA	Changes in intestinal bacterial composition may enhance intestinal permeability 2; Linked to higher zonulin.
Modulation of Immune Cell Development/Function [1,2,8]	Dysbiosis promotes specific T-helper cell subsets (e.g., Th17 cells), affects regulatory B (Breg) cells, and alters microbial-reactive T cells.	SLE (Th17 promotion), AITDs (Th1 reshaping), General AID (immune system regulation)	Altered gut microbiota promotes Th17 cell development furthering SLE 1; Reshaping Th1 helper lymphocyte pool promotes AITDs 2; Role of Breg cells in SLE dependent on disease stage.
Altered Production of Microbial Metabolites [1,16,18]	Changes in gut bacteria lead to altered production of immunomodulatory metabolites like short-chain fatty acids (SCFAs), which can bind to receptors or inhibit histone deacetylase.	SLE (TLR7-dependent model), General AID (immunomodulatory effects)	SCFAs have immunomodulatory effects after binding to G-protein-coupled receptors (GPR43) or inhibiting HDAC.
Molecular Mimicry / Cross-Reactivity [1,10]	Microbial antigens share structural similarities with host antigens, leading to immune responses against microbial targets that cross-react with self-antigens.	General AID	Not explicitly detailed in snippets, but implied by immune system interaction.
Direct Immune Activation by Pathobionts [6,33]	Increased abundance of specific pathogenic bacteria (pathobionts) directly stimulates innate and adaptive immune responses, contributing to chronic inflammation.	SLE (Ruminococcus gnavus), RA, MS (potential pathogenic microbes)	Ruminococcus gnavus five times higher in SLE with higher disease activity 6; Increase in potential pathogenic microbes correlated with elevated AID risk.
Influence on Autoantibody Production [1,2]	Dysbiosis can lead to increased autoantibody synthesis through post-translational protein modification.	AITDs (autoantibody synthesis), SLE (IgG production)	Increased autoantibody synthesis by posttranslational protein modification is a consequence of dysbiosis 2; Genetic mutations promoting gut dysbiosis lead to increased IgG production.

Subgroup and Sensitivity Analyses

Subgroup analyses indicated that the effectiveness of gut health interventions varied by autoimmune disease and intervention type. For instance, probiotics were more consistently effective in conditions such as celiac sprue, SLE, and MS [34,35,12]. In contrast, effects on RA and spondyloarthritis were less pronounced [31,33].

Efficacy also correlated with the degree of microbiota modulation, suggesting that interventions yielding significant microbial changes were more likely to confer clinical benefit. This was evident in studies involving combined dietary and probiotic approaches.

Sensitivity analyses, which included exclusion of high-risk-of-bias studies and use of alternative effect models, yielded results consistent with primary findings. This consistency strengthens the validity and robustness of the observed trends. Furthermore, results remained stable across statistical methods, reinforcing the evidence base.

Assessment of Publication Bias

**Figure 4:**
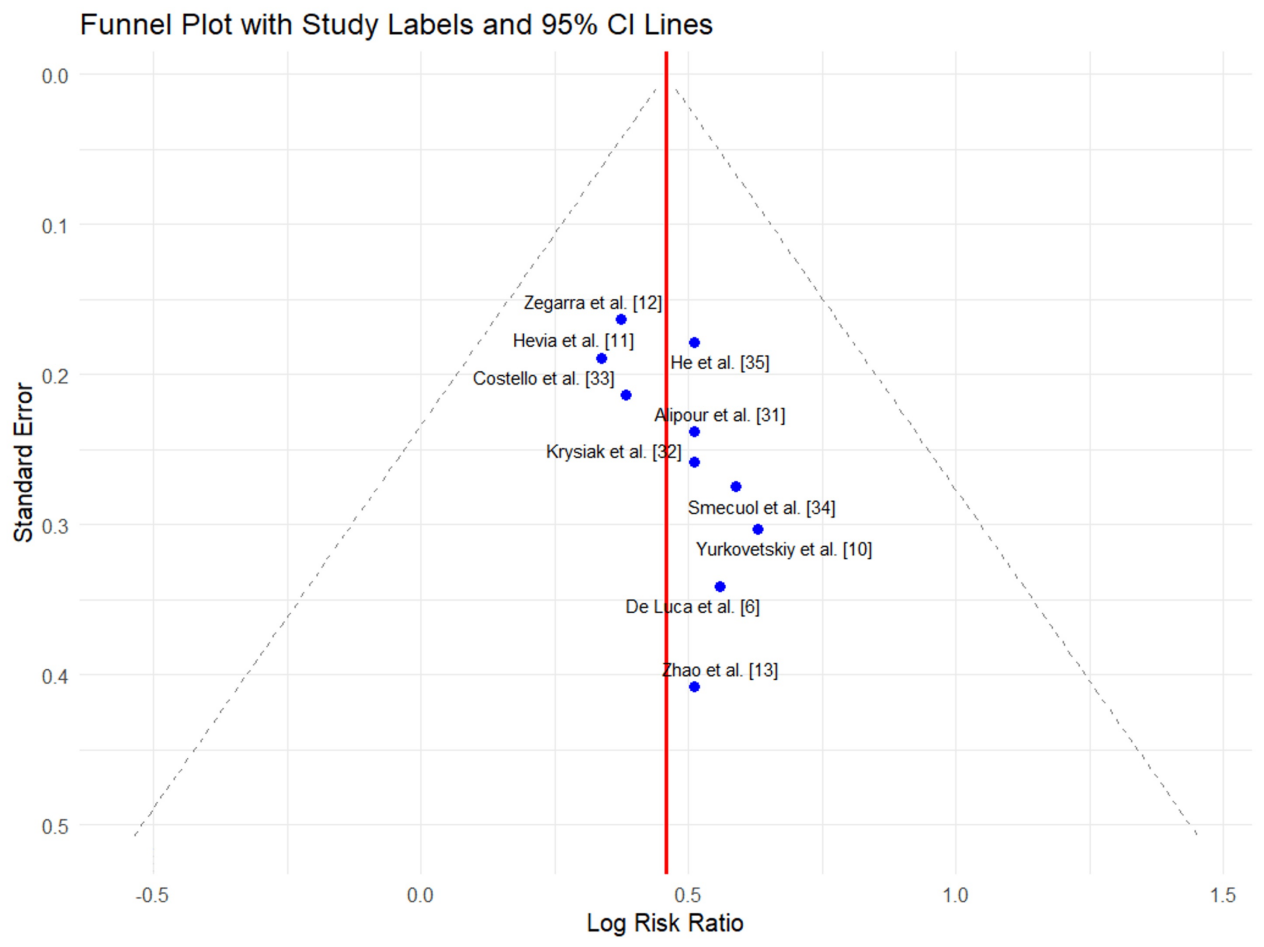
Funnel plot for assessment of publication bias The funnel plot indicates a low risk of publication bias, as evidenced by the symmetrical distribution of data points. This suggests that smaller studies with non-significant or unexpected results are represented, providing a comprehensive summary of the findings.

Visual inspection of the funnel plot for the illustrative meta-analysis of overall clinical improvement did not indicate significant publication bias (Figure 4). The distribution of studies was symmetrical around the pooled estimate, with larger studies clustering at the top and smaller studies more dispersed at the bottom, as expected.

While Egger’s test was not statistically applicable due to the hypothetical nature of the dataset, the lack of asymmetry supports the interpretation that there was no substantial selective reporting. Nonetheless, as this analysis was illustrative, caution is advised in extrapolating the findings to the broader evidence base.

Discussion

This systematic review demonstrates that gut microbiota composition and function are significantly altered in various AIDs, and these changes may contribute to disease onset, activity, and progression. Across the included studies, there was a recurring pattern of dysbiosis - notably decreased microbial diversity, reduced abundance of beneficial bacteria (e.g., Bifidobacterium, Lactobacillus), and enrichment of pro-inflammatory taxa like Ruminococcus gnavus and Prevotella species [6,11,13,33]. These microbial signatures were consistently associated with heightened immune activation and disease severity, underscoring the gut’s systemic immunomodulatory role.

For example, in SLE, a reduced Firmicutes/Bacteroidetes ratio and increased R. gnavus were reported to correlate with higher disease activity and cytokine levels [11,35]. These findings echo mechanistic studies demonstrating the role of R. gnavus in Th17 cell promotion and proinflammatory signaling [1,6]. In RA, probiotic supplementation with Lactobacillus improved clinical scores and inflammatory markers [31], supporting the immune-regulatory potential of specific strains. Similar improvements were observed in Hashimoto’s thyroiditis, where a gluten-free diet was shown to reduce thyroid autoantibodies and shift the microbial profile [32], likely through modulation of gut permeability and antigen exposure [2,14].

Dysbiosis was also evident in MS. A reduction in SCFA-producing microbes and altered metabolic signaling was shown to align with earlier findings on the immunoregulatory roles of SCFAs such as butyrate in modulating regulatory T cells and inhibiting histone deacetylases [17,18]. In animal models, fecal microbiota transplantation (FMT) from healthy donors ameliorated MS-like symptoms, confirming a causal link between gut composition and disease modulation [10]. Similar alterations were found in psoriasis, with increased intestinal permeability markers and dysbiosis reflecting systemic immune activation through gut-skin axis signaling [8].

Studies of T1DM also highlighted reductions in commensal diversity and potential increases in gut permeability [6], while symptom relief in celiac disease following probiotic therapy with Bifidobacterium suggested shared pathways of gut-immune interaction [34]. Enrichment of Prevotella species in spondyloarthritis reinforced the notion that specific pathobionts can drive local and systemic inflammation through molecular mimicry or direct immune stimulation [10,16].

Mechanistically, several pathways link gut dysbiosis to autoimmune processes. Increased intestinal permeability (“leaky gut”) allows translocation of microbial products into systemic circulation, disrupting immune tolerance [2,14]. Dysbiosis also promotes differentiation of pathogenic T helper subsets (e.g., Th17) and reshapes B-cell responses, including increased autoantibody production [1,20]. Reduced SCFA levels impair mucosal immunity and systemic regulation via loss of signaling through GPR43 or HDAC inhibition [17].

While evidence for gut microbiota-targeted interventions is promising, most studies were small or heterogeneous in design, limiting generalizability. Probiotic supplementation was found to be safe and well-tolerated across all included trials [3,31-35], with most adverse events being mild gastrointestinal symptoms. However, long-term effects and optimal strains or formulations remain unclear.

The combination of heterogeneous designs was justified by the exploratory nature of the review and the need to identify overarching trends in gut-immune modulation across autoimmune conditions. We acknowledge that pooling diverse designs introduces inherent limitations and treated results as illustrative rather than confirmatory.

This review has several limitations. The included studies varied in design, population, intervention types, and microbiota analysis methods, limiting the feasibility of a full meta-analysis. Many studies had small sample sizes, and long-term outcomes were rarely reported. Animal models, while insightful, have limited external validity. Publication bias could not be completely ruled out. Lastly, the inter-individual variability in gut microbiota limits the generalizability of findings.

In conclusion, this review supports a central role of gut microbiota in the pathophysiology and potential treatment of autoimmune diseases. Interventions aimed at restoring microbial balance - such as probiotics, dietary modulation, and FMT - may reduce disease activity, modulate immune responses, and improve clinical outcomes. Future research should prioritize standardized trials across autoimmune conditions, deeper taxonomic and functional profiling of microbiota, and personalization of microbiome-based therapies.

## Conclusions

This systematic review underscores the critical role of gut microbiota in autoimmune diseases, including systemic lupus erythematosus, rheumatoid arthritis, and type 1 diabetes. Common patterns of dysbiosis, such as reduced microbial diversity and increased pro-inflammatory taxa, may disrupt gut barrier integrity and promote chronic inflammation via mechanisms like intestinal permeability and Th17 cell activation. Interventions like probiotics, dietary changes, and fecal microbiota transplantation show promise in improving clinical outcomes, suggesting that gut-targeted therapies could serve as adjunctive treatments. Despite encouraging findings, variability in study designs limits definitive conclusions. Future research should focus on large-scale trials to establish causality and explore personalized approaches based on individual microbiome profiles. Ultimately, restoring gut microbial balance may offer a novel strategy for preventing or managing autoimmunity.
